# Spoilage of tilapia by Pseudomonas putida with different adhesion abilities

**DOI:** 10.1016/j.crfs.2022.04.002

**Published:** 2022-04-11

**Authors:** Wen Zhang, Yunru Wei, Xilin Jin, Xucong Lv, Zhibin Liu, Li Ni

**Affiliations:** Institute of Food Science and Technology, College of Biological Science and Technology, Fuzhou University, Fuzhou, 350108, China

**Keywords:** *Pseudomonas putida*, Adhesion ability, Spoilage, Intestinal flora, Volatile components, TVB-N, Total Volatile Base Nitrogen, SPEM-GC/MS, Solid Phase Microextraction-Gas chromatography/Mass spectrometer

## Abstract

Four *Pseudomonas putida* strains isolated from spoiled tilapia were divided into three adhesion abilities—high, medium, and low—by an in vitro mucus model. Four strains had no significant difference in spoilage ability to the inoculated fish fillets. However, according to the in vivo experiment, the spoilage caused by the four *P.putida* was positively correlated with their adhesion abilities. High adhesion strains not only caused more TVB-N in chilled fish, but also activated the spoilage activity of intestinal flora. The diversity of intestinal flora and the changes in volatile components in fish were detected by high-throughput sequencing and SPME-GC/MS. The strains with high adhesion abilities significantly changed the intestinal flora, which led to a significant increase in low-grade aldehydes, indole, and esters in flesh of fish, as well as the production of a fishy and pungent odor. The intestinal adhesion ability of spoilage bacteria was considered the key factor in spoilage of chilled fish.

## Highlights

• A positive correlation between the intestinal adhesion ability of *P.putida* and the spoilage ability in vivo.
• *P.putida* affected the intestinal microflora and led to increase in fishy and pungent odor.
• The intestinal adhesion ability of *P.putida* was considered as a key factor in spoilage.

## Graphical abstract

**Figure:**
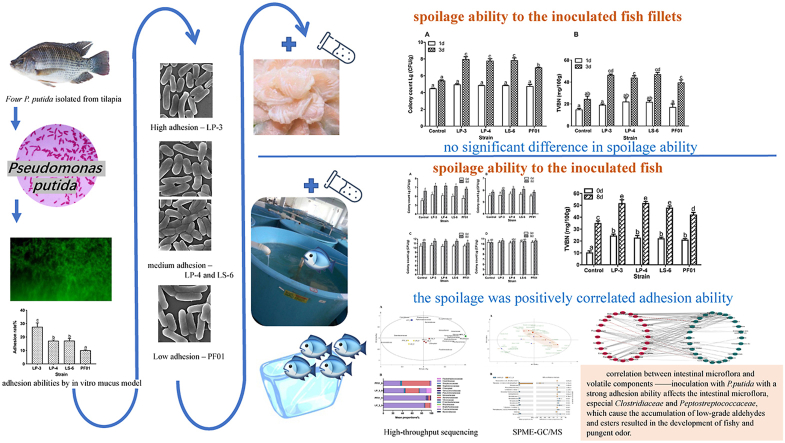

## 1 Introduction

In the production, distribution and storage of meat products, the loss caused by microbial accounts for about 20% of the total loss of food. (FAO, 2011; Höll et al., 2016). Fish products are extremely perishable and sensitive to microbial growth, while some microflora are suitable for survival and take part in the spoilage process, producing spoilage odors and off-flavor metabolites (Gram and Dalgaard, 2002).

*Pseudomonas* spp. are Gram-negative aerobic bacteria and very common in fresh foods, the genus was subdivided into five rRNA similarity groups based on DNA-DNA hybridization studies. The species that are closely related to the spoilage of meat products are in group I, include *Pseudomonas fragi*, *Pseudomonas fluorescens*, and *Pseudomonas lundensis* and others (Ercolini et al., 2010; Mellor et al., 2011; Liu et al., 2015). *P.putida* is considered the main component of these spoilage microbial with an incidence between 56.7% and 79.0% on spoilt meat (Tryfinopoulou et al., 2002; De Jonghe et al., 2011; Doulgeraki and Nychas, 2013; Mohareb et al., 2015). Markers of microbial spoilage include total number of bacteria, chemical indicators including total volatile basic nitrogen (TVB-N), trimethylamine (TMA), and volatile organic content (VOC)and enzyme-producing capacity (Jaffrès et al., 2011; Ge et al., 2017; Parlapani et al., 2017; Silbande et al., 2018; Papadopoulou et al., 2020; Huang et al., 2021). Recent studies discussed the molecular mechanism of spoilage-related enzymes and quorum sensing (Zhu et al., 2016; Wang et al., 2021).

Our previous studies found that the adhesion and spoilage ability of *Shewanella* spp. in mariculture large yellow croaker in vivo and in vitro were significantly higher than those of *Pseudomonas* spp. (Zhang et al., 2019, Zhang et al., 2019). Therefore, we speculated that adhesion ability plays an important role in fish spoilage. Some *Pseudomonas* spp. were isolated from spoiled tilapia. By comparing the adhesion ability and spoilage ability in vitro, four *Pseudomonas putida* strains were selected for in vivo test. Combined with the intestinal flora diversity and volatile compounds of fish, the role of adhesion of spoilage bacteria in the spoilage of chilled tilapia was discussed. The results of this study may provide a new strategy for further revealing the spoilage process and the mechanism of spoilage bacteria. The results may also provide a theoretical reference for the development of preservatives.

## 2 Materials and methods

### 2.1 Materials and strains

*Pseudomonas* spp*.* were isolated from spoiled tilapia, through 16 S rRNA construct a phylogenetic tree and physiological and biochemical identification, they were identified as *P.putida* and registered in GenBank, as shown in Table A. Four strains of *P.putida* were LP-3, LP-4, LS-6 and PF01. The fresh tilapias were donated by Fuzhou Aquaculture Institute.

### 2.2 Bacterial adhesion ability in vitro

Mucus was prepared using the method published by Chen et al. (2008)**,** with slight modifications. The intestinal mucus of tilapia was harvested by scraping off the inner surface of the intestines with a spatula to remove the mucous gel layer covering the intestine. Finally, the mucus was homogenized in sterile 0.01 mol L1 PBS and centrifuged twice at 10,000 g for 30 min at 4 °C to remove particulate matter. The supernatant was filtered through 0.22-μm filters. The mucus samples were adjusted to 0.5 mg protein mL^−1^ with sterile 0.01 mol L^−1^ PBS and stored at −20 °C until use. The protein concentration was determined by the Lowry method (Hartree, 1972).

FITC staining was performed according to a previously published method (Vinderola et al., 2004). Fresh cultures of each strain were cultured overnight at 28 °C. Then, bacteria were harvested by centrifugation at 10,000 g for 10 min at 4 °C and washed twice with 0.01 mol L^−1^ PBS (pH 7.4). Cells were labeled with fluorescein isothiocyanate (FITC; Sigma, St Louis, MO, USA, 0.2 mg mL^−1^) in PBS and incubated for 1.5 h at 30 °C in the dark. Labeled bacteria were washed several times with PBS solution to remove unincorporated FITC. The final pellet was resuspended in PBS to a concentration of 1 × 10^8^ CFU mL^−1^ and stored at −20 °C in the dark.

Mucus amounting to 150 μL was added to black 96-well plates and stored overnight at 4 °C. The residual mucus was washed twice with 200 μL of sterile 0.01 mol L^−1^ PBS. FITC-labeled bacterial suspension (150 μL) was added to the wells and incubated at 4 °C for 90 min. The non-adhered bacteria were flushed twice with sterile physiological saline. The adhered bacteria were released and lysed with 150 μL of 1% SDS (0.1 mol L^−1^ NaOH) solution at 60 °C for 1 h. Fluorescence was measured with a multiscan fluorometer (SpectraMax i3+MiniMax, Molecular Devices, USA) at λex 495 nm and λem 525 nm. Negative controls of labeled bacteria were used to calculate the percentage of adhesion. This percentage was expressed as the percentage of fluorescence recovered after attachment to mucus relative to the initial fluorescence of the bacterial suspension added to the wells (Equation (1)).

(1) $$Adhesionrate\%=\left(FI_{2}/FI_{1}\right)\times 100$$

where FI_2_ and FI_1_ are the fluorescence intensities of the experimental group and the FITC-labeled pure bacterial suspension, respectively.

### 2.3 Spoilage potential evaluation

The fresh tilapia (approx. 500 g) were transported to the laboratory on ice from Fuzhou Aquaculture Institute, China, within 6 h of fishing. They were subsequently scaled; their gills and guts were removed. The fish were cleaned and filleted (2.0 ± 0.1 g each after cleaned). Then, the fillets were washed thoroughly with sterile water, placed in a sterile Petri dish, and irradiated with ultraviolet light for 20 min. Sterile fish fillets were soaked in a bacterial suspension of *P.putida* (approx. 10^8^ CFU mL^−1)^ for 20 s, drained, and placed in a 50-mL sterile centrifugal tube. The tube was stored at 4 °C. Sterile fish fillets without inoculated bacteria were used as a blank control.

A fish sample (2 g) was homogenized with 18 mL of sterile saline solution for 2 min. Then, genomic DNA was extracted and total number of bacteria was determined by qPCR. The fish fillets were homogenized. TVB-N was determined following the method described by Malle and Poumeyrol (1989).

The SPME method published by Mikš-Krajnik et al. (2016) was used for reference, with slight modifications. Samples (2.00 g) were transferred to a 15-mL glass vial, followed by the addition of 5.00-mL NaCl and 2 μg/mL three methyl pyridine (TMP). After equilibration at 60 °C for 3 min, VOCs were extracted with an aging SPME fiber for 30 min. Finally, the fiber was inserted into the GC injection port immediately and analyzed at 250 °C for 3 min. The absolute concentration was obtained by calculating the ratio of the measured volatiles to the peak area of TMP, assuming that the absolute correction factor of each volatile was 1.0 (Equation (2)).

(2) Volatilecomponentconcentration(μg/g)=((Ai/A)×0.4)/2

where *A*_*i*_ is the peak area of each volatile component; *A* is the peak area of the volatile component TMP; 0.4 is the amount of TMP added; and 2 is the mass of the sample (g).

### 2.4 Feeding experiment

Two hundred tilapia, with an average body weight of 20 g, were randomly divided into a control group or a treatment group supplemented with *P.putida* (either the LP-3 group, LP-4 group, LS-6 group, or PF01 group). There were three replicates per group, and 20 tilapia per replicate. Feed was supplied twice a day (09:30 and 17:30 h). During the experiment, 0.01% bacterial suspension with water volume fraction was added daily, the final bacterial content was approximately 1.0 × 10^4^ CFU/mL. The cultivation water was fresh water, with a temperature range of 22 °C–24 °C. Approximately 30% of cultivation water was changed daily, and unconsumed feed and fish feces were purged. After one week of adaptation with basal feed, the feeding experiment began and lasted one week.

After 24 h of fasting at the end of the experiment, all fish were caught and immediately stored at 4 °C. At storage for 1 d and 3 d, the fish were washed with sterile water to reduce contamination by commensal bacteria. Digestive tracts were aseptically removed and homogenized in a sterile homogenization bag. The bags were stored at −20 °C for DNA extraction. The fish were scaled; their gills and guts were removed. The fish were cleaned, filleted (2.0 ± 0.1 g each after cleaned), and stored at −20 °C for the TVB-N test.

### 2.5 DNA extraction, qPCR reaction, and high-throughput sequencing analysis

Total DNA was extracted from the intestines using a fecal genomic DNA extraction kit and bacterial genomic DNA extraction kit (Tiangen, China). The qPCR reaction consisted of 2.0 μL of 10-fold diluted DNA, 10 μL of SYBR Green, and 0.8 μL of each primer (10 μM) in a total volume of 20 μL. The PCR program comprised 45 cycles at 95 °C (35 s), 60 °C (30 s), and 72 °C (30 s), followed by melt curve generation. Melt curves were analyzed to check the specificity of amplification. Gene-specific primers were designed as:

Pseudomonas, Pse-F, CTGCATCATGGCCGGTGACAACATTT, Pse-R, GTCGCATGGCTGTCGGTCTTCAGATC, were used to qPCR for *Pseudomonas*.

Bacterial primers, 27-F, AGAGTTTGATCCTGGCTCAG, 1492-R, GGTTACCTTGTTACGACTT, were used to qPCR for total number of bacteria.

Bacterial primers 341-F (50-CCT AYG GGR BGC ASC AG-30) and 806-R (50-GGA CTA CNN GGG TAT CTA AT-30) were used to amplify the V3–V4 region of bacterial 16SrRNA genes. The sequencing library of bacterial 16 S rRNA genes was generated for high-throughput sequencing, employing the TruSeqfi DNA PCR-Free Sample Preparation Kit (Illumina, San Diego, CA, USA). Next, the library was sequenced on an Illumina HiSeq2500 platform by Novogene Bioinformatics Technology Co., Ltd. (Beijing, China).

### 2.6 Statistical analysis

The results were expressed as mean ± standard deviation, and the probability value of P < 0.05 was considered significant. Significance analysis for all graphs was obtained by comparative analysis within and between groups. An LSD test with Statistical Package for Social Sciences 24.0 (SPSS for Windows, SPSS Inc., Chicago, IL, USA) was used for significance analysis. GraphPad Prism 8.0 (GraphPad Software Inc., San Diego, CA, USA) was used to make the graphs. Bidirectional orthogonal partial least squares (O2PLS) modeling was used to elucidate the relationships between functional microbiota and flavor formation; PLS-DA was performed using SIMCA-14.1 software (UMETRICS, Malmo, Sweden). STAMP (Statistical Analysis of Metagenomic Profiles) was used to perform difference analysis.

## 3 Results and discussion

### 3.1 In vitro adhesion and spoilage by *Pseudomonas putida*

#### 3.1.1 In vitro mucus adhesion ability of *P.putida*

**Fig. 1:**
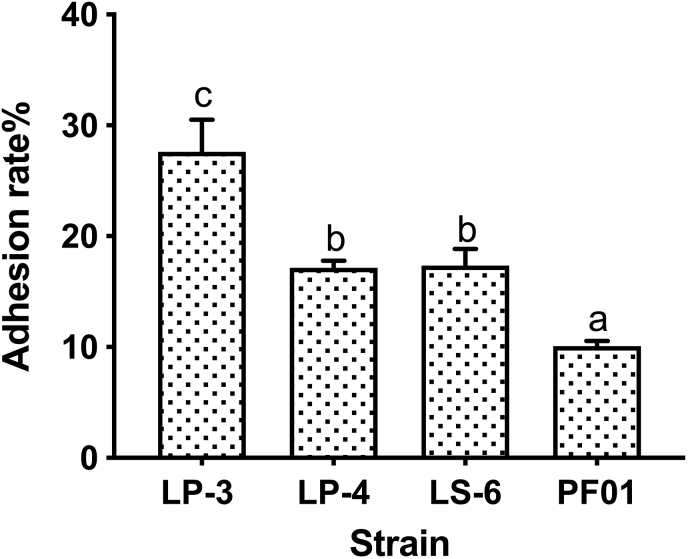
Mucus adhesion ability of *P.putida*. Data was expressed as mean ± standard deviations (n = 4), and significance was measured by using the 2way-ANOVA.Different lowercase letters indicate significant differences at 0.05 level (p < 0.05).

As shown in Fig. 1, the adhesion abilities of the four *P.putida* strains in fish intestinal mucus were divided into high adhesion strain LP-3, medium adhesion strains LP-4 and LS-6, and low adhesion strain PF01 by an in vitro mucus model.

In the process of bacterial adhesion to the host, it first contacts the mucus layer and then adheres to intestinal epithelial cells (Tassell and Miller, 2011).Therefore, in vitro mucus model is an effective model to evaluate bacterial adhesion. In vitro mucus model was used to measure the adhesion of candidate probiotic lactic acid bacteria (LAB) to carp intestinal mucus and it was found that there was a strong correlation between mucus adhesion in vitro and colonization ability in vivo (Sugimura et al., 2011).We found the difference of adhesion ability of four strains of *P.putida* in vitro and speculated it would lead to differences in fish spoilage.

#### 3.1.2 Analysis of spoilage ability in vitro

**Fig. 2:**
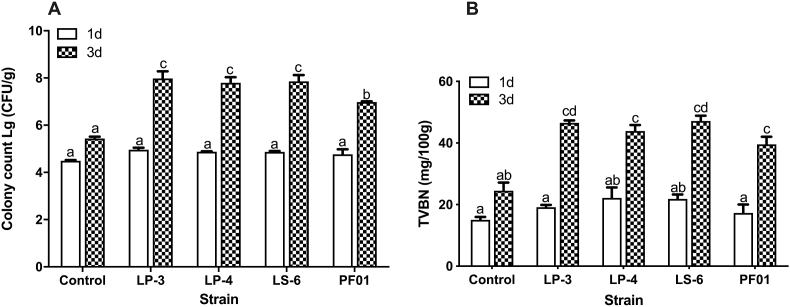
In vitro evaluation of the spoilage potential of *P.putida* LP-3, LP-4, LS-6 and PF01 by inoculating cultures on fish fillets and storing at 4 °C. (A) Total number of bacteria; (B) TVB-N value. Data was expressed as mean ± standard deviations (n = 3), and significance was measured by using the 2way-ANOVA. Different lowercase letters indicate significant differences at 0.05 level (p < 0.05).

The four strains of *P.putida* were inoculated in fresh, sterile tilapia fillets, and the changes in the total number of bacteria and TVB-N during cold storage were measured. The growth of the four strains was similar, except that PF01 grew slowly in a later stage (3 d) (Fig. 2A). *P. putida* LS-6 produced the highest TVB-N, followed by LP-3 and LP-4, and then finally PF01 (Fig. 2B). However, there was no significant difference in spoilage ability (p > 0.05).

TVB-N, TMA (Trimethylamine) and K value are often used to indicate the spoilage ability (Park et al., 2021, Peleg, 2016). Research showed that there was a correlation between adhesion and biofilm formation (Garrett et al., 2008; Muhammad et al., 2020). For example, flagella can promote adhesion, colonization, and biofilm formation, resulting in a large number of bacteria (Conrad, 2012; Bruzaud et al., 2015). On the contrary, the adhesion ability of PF01 was weak, which made it difficult to form biofilm on fish fillets, so the total number of bacteria was less. However, it could be seen that PF01 shown individual strong spoilage ability.

### 3.2 In vivo adhesion and spoilage of *Pseudomonas putida*

#### 3.2.1 *P.putida* colonization in fish intestine and muscle

**Fig. 3:**
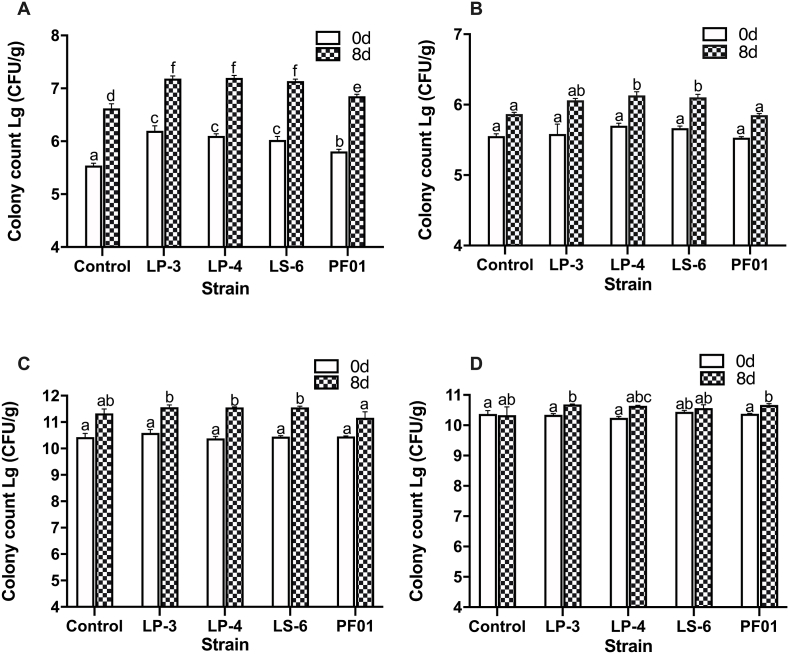
qPCR analysis of bacterial growth in the intestine and flesh of tilapia during storage at 4 °C. (A) *Pseudomonas* spp. in intestine; (B) *Pseudomonas* spp. in flesh; (C) Total number of bacteria in intestine; (D) Total number of bacteria in flesh. Data was expressed as mean ± standard deviations (n = 3), and significance was measured by using the 2way-ANOVA. Different lowercase letters indicate significant differences at 0.05 level (p < 0.05).

After feeding tilapia with four *P.putida* strains for one week, the number of *Pseudomonas* and total bacteria in intestine and flesh of tilapia were determined by qPCR at 0 d and 8 d of storage to characterize the adhesion of *Pseudomonas* in vivo. Compared with the control, four *P.putida* strains adhered to the fish intestines, and the adhesion of PF01 in the intestine was significantly lower than that of other strains (as shown in Fig. 3A, at 0 d). The results showed that *P.putida* could adhere to fish intestines, and PF01 had the lowest adhesion in vivo and in vitro. After eight days of cold storage, the number of *Pseudomonas* in intestine was significantly higher than that in the control group, and there was no significant difference except that PF01 group was slightly lower. It showed that *Pseudomonas* has been fully bred during storage. There was no difference in the number of *Pseudomonas* in the flesh at 0 day, but the count of *Pseudomonas* in the flesh of PF01 was lower than other treatment groups at 8 d. It was consistent with adhesion ability in vitro mucus model. Because the adhesion ability is related to the bacterial motility, the diffusion ability of bacteria with low adhesion is weak (Nejidat et al., 2008; Navarrete et al., 2019; Suchanek et al., 2020). Therefore, *Pseudomonas* was also the lowest in the flesh of PF01. There was no significant difference in the total number of bacteria among the groups, which indicated that the addition of *P.putida* did not affect the total number of bacteria.

#### 3.2.2 Quantitative analysis of TVB-N production and spoilage ability of *P.putida* in fish

**Fig. 4:**
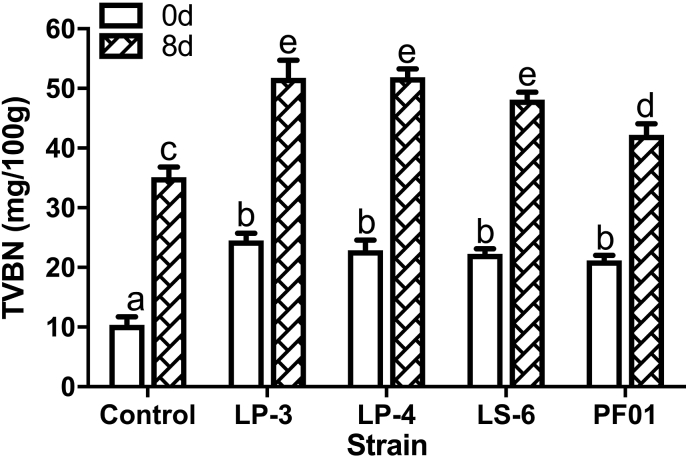
TVB-N value of tilapia inoculated with different *P.putida* and stored at 4 °C. Data was expressed as mean ± standard deviations (n = 3), and significance was measured by using the 2way-ANOVA. Different lowercase letters indicate significant differences at 0.05 level (p < 0.05).

The tilapia inoculated with *P.putida* were stored at 4 °C. TVB-N in the flesh was measured at 0 d and 8 d. As shown in Fig. 4, TVB-N in the treatment group was significantly higher than that in the control group at 0 d (P < 0.05). There was no significant difference in TVB-N content between LP-3, LP-,4 and LS-6. The concentration in all samples was approximately 24.59 mg/100 g, and the content of TVB-N produced in PF01 group was lower than the other three groups. TVB-N in each group exceeded 30 mg/100 g at 8 d, and TVB-N concentration in LP-3 and LP-4 was the highest, followed by LS-6, and then PF01. Thus, TVB-N content is positively correlated with adhesion ability.

The spoilage ability of spoilage bacteria is related to the activity of protease and growth ability(Bekker et al., 2015, Shao et al., 2021). Our study found that strains with similar spoilage ability on fish fillets have different spoilage potential in vivo due to their adhesion and colonization ability.

#### 3.2.3 Intestinal flora diversity based on high-throughput sequencing results

**Fig. 5:**
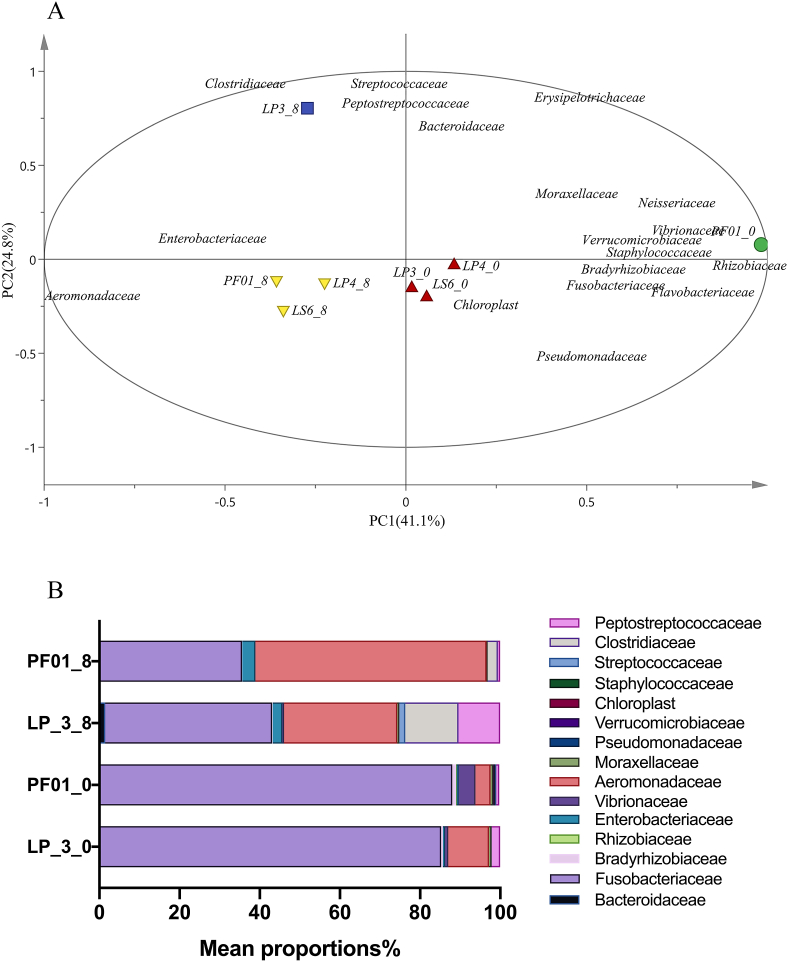
(A) Principal component analysis of intestinal flora of tilapia inoculated with *P.putida* and stored at 4 °C for 0 d and 8 d. (B) Intestinal flora composition of tilapia inoculated with *P.putida* LP-3 and PF01 and stored at 4 °C for 0 d and 8 d.

The diversity of intestinal flora of tilapia stored for 0 and 8 d was determined by high-throughput sequencing. Principal component analysis (PCA) showed that, compared with the other three groups, the intestinal flora structure of PF01 group with weak adhesion was significantly different in the initial stage (0 d), while LP-3 with strong adhesion was significantly different from the other three groups in the spoilage stage (8 d). As shown in Fig. 5A, the medium and high adhesion groups (LP-3, LP-4, and LS-6) were grouped together, and *Pseudomonas* is the main bacterium in the initial storage stage indicating that *P.putida* exhibited high adhesion and had strong colonization ability in the fish intestinal tract. The intestinal flora structure of PF01 at 0 d was the most different from that of LP-3 at 8 d, as shown in Fig. 5B, the intestinal flora in PF01 at 0 d exhibited weak adhesion ability mainly consisted of *Vibrionaceae*, *Rhizobiaceae,* and *Moraxellaceae* except *Fusobacteriaceae,* and the intestinal flora in LP-3 at 8 d exhibited high adhesion ability and mainly consisted of *Stretocococcaceae*, *Bacteroidaceae*, *Clostridiaceae*, and *Peptotrecoccaceae*. *Fusobacteriaceae* decreased during storage. *Aeromonas* increased during storage and had advantages in low adhesion PF01. *Clostridiaceae* and *Peptostreptococcaceae* increased significantly in high adhesion LP-3.

#### 3.2.4 Volatile components in spoilage fish

Aquatic products are affected by the degradation of endogenous enzymes, lipid oxidation, microbial reproduction, and environmental factors, which reduce freshness and generate peculiar odors. The volatile components in tilapia were determined by SPME-GC-MS at 0 d and 8 d storage. Forty-eight volatile substances were detected, including alkanes, alcohols, aldehydes, ketones, esters, and aromatic compounds, as shown in Table B.

**Fig. 6:**
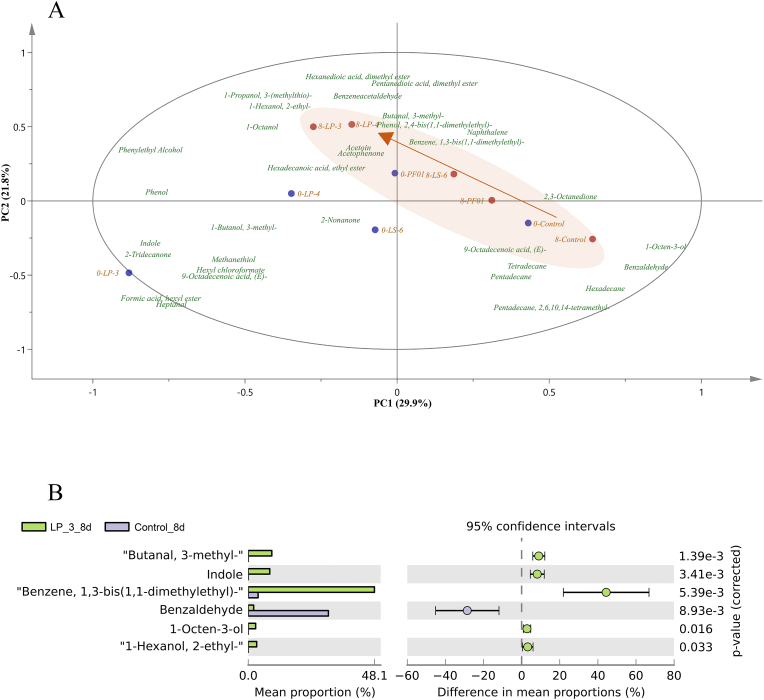
(A) Principal component analysis of VOCs (VIP value > 1) for tilapia inoculated with *P.putida* and stored at 4 °C for 0 d and 8 d. (B) Differences in volatile components in the flesh of tilapia from the LP-3 and control groups at 8 d. Data was expressed as mean proportion (n = 3), and significance was measured by using the Welch's *t*-test (Two-sided).

Content of volatile compounds was subjected to partial least squares discriminant analysis (PLS-DA) and calculated the value of variable importance in projection (VIP). 32 key volatile components (VIP >1.0) that are associated with the process of spoilage were screened. PCA analysis of the volatile compounds is shown in Fig. 6A. Compared with the control, the addition of *P. putida* changed the flavor of fish at both the starting point and the end point of spoilage. At the beginning of storage (0 d), LP-3 was the farthest away from the control group, and LP-4, LS-6, and PF01 were close to the control group. After 8 d of storage, LP-3 and LP-4 were the farthest away from the control group, with similar odor characteristics, while LS-6 and PF01 were close to the control group. The results show that the higher the adhesion ability of *Pseudomonas putida*, the easier it was to change the flavor profile of fish (as shown by the arrow in Fig. 6A).The aldehydes in the *Pseudomonas* group increased significantly, include low-grade aldehydes, such as benzeneacetaldehyde and 3-methyl-butanaland hexanal. *Pseudomonas* spp. (mainly *Pseudomonas fragi* and *Pseudomonas fluorescens*) can produce many low-grade aldehydes (Boziaris and Parlapani, 2017). Studies have shown that low-grade aldehydes, such as hexanal, have unpleasant grassy and pungent odors, and are commonly found in freshwater fish (Hirano et al., 1992; Thiansilakul et al., 2010; Ma et al., 2020).

It was shown that butanal, indole and benzene were the differential volatile components of LP -3 on at 8 d (Fig. 6B). Indole was detected in the *Pseudomonas* treatment groups, especially in LP-3 group, but it was not detected in the control group. The content of indole was high in spoiled fish, and indole contributes to the pungent smell (Yang et al., 2011). Benzene, 1,3-bis(1,1-dimethylethyl)-, which is considered a marker of irradiated food, possibly because methyl radicals continue to bind to xylene radical during irradiation (Kim et al., 2005; Caja et al., 2008).It suggested that spoilage bacteria accelerated the oxidation of fish.

In general, the addition of high-adhesion *P.putida* accelerated the spoilage of fresh fish. The accumulation of low-grade aldehydes, indole, and esters resulted in the development of fishy and pungent odors, especially in the LP-3 and LP-4 groups.

**Fig. 7:**
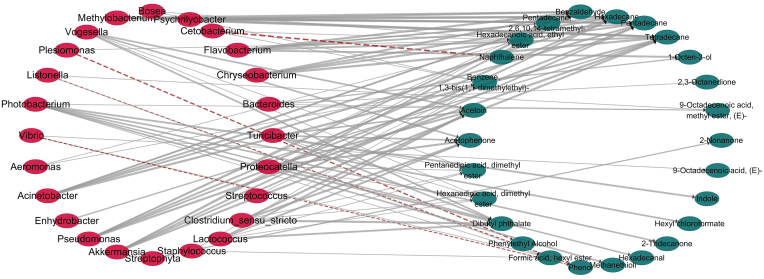
Network model of the correlation between microbial genera and volatile compounds. The grey line represents positive correlation, the red line represents negative correlation. The thicker the lines, the stronger the correlation. Correlation coefficients were obtained by Pearson's correlation analysis. (For interpretation of the references to colour in this figure legend, the reader is referred to the Web version of this article.)

The O2PLS method was used to investigate the correlation between microorganisms and volatile compounds of spoiled fish in different *Pseudomonas* treatment groups. A network model for the correlation between microbial genera and volatile compounds was drawn based on Cytospace software. Twenty-four bacterial genera were highly correlated (|ρ| > 0.6) with volatile compounds (Fig. 7), and most of them had a positive correlation with volatile compounds, except *Plesiomonas*, *Cetobacterium*, *Listonella* and *Bosea*. This indicated that the change in microbial flora is accompanied by the formation of volatile compounds.

Combined with the results of high-throughput sequencing, *Clostridaceae* and *Bacteroidaceae* were the dominant families in the LP-3 treatment group, while *Clostridium* was highly positively correlated with acid esters (pentanedioic acid, dimethyl ester, hexanedioic acid, dimethyl ester, dibutyl phthalate), which are usually the products of the esterification of carboxylic acids and alcohols generated by lipid oxidation. Many scholars believe that lipid oxidation is an important cause of fishy odors (Feng et al., 2012; Shahidi, 2016). *Bacteroides* has a highly positive correlation with 2,3-octanedione, a volatile substance related to lipid oxidation with an unpleasant grassy smell (Gomez et al., 2020). Study showed that *Clostridaceae* and *Bacteroidaceae*can promote β-oxidation processes, and β-oxidation leads to lipid oxidation (Coma et al., 2016). Therefore, we speculate that these bacteria promoted lipid oxidation and produced more ester acids with fishy aromas.

The above conclusions prove that *P.putida* with its high adhesion ability, not only has a strong spoilage factor, but can also lead to more serious spoilage by affecting the structure of the microflora.

## 4 Conclusion

Spoilage bacteria adhere and colonize on fish. The breed of spoilage bacteria led to fish spoilage. Therefore, this study aims to reveal the relationship between bacterial adhesion and spoilage. Four strains of *P.putida* isolated from tilapia showed no significant difference in their spoilage ability in fish fillet spoilage experiment. Through tilapia feeding experiment, four *P.putida* strains were added to the water and adhere to the fish, so as to compare their spoilage ability in vivo. The results confirmed a positive correlation between the intestinal adhesion ability of *P.putida* and the spoilage ability. Higher adhesion, more TVB-N. Moreover, the correlation between intestinal microflora and volatile components proves that inoculation with *P.putida* with a strong adhesion ability affects the intestinal microflora, especial *Clostridiaceae* and *Peptostreptococcaceae*, which cause the accumulation of low-grade aldehydes, indole, and esters resulted in the development of fishy and pungent odor. Therefore, it was shown in our work that the adhesion ability of *P.putida* was the premise of spoilage in fish, and the adhesion ability could be used as an important index of spoilage ability, especially for chilled whole fish.

## Funding

This work was supported by the Science Foundation of the Fujian Province, China (No. 2020J01487) and the Science and Technology Project of Fuzhou (No. 2020-GX-13).

## Ethical approval

All animal experiments comply with the ARRIVE guidelines and be carried out in accordance with the National Research Council's Guide for the Care and Use of Laboratory Animals.

## CRediT authorship contribution statement

**Wen Zhang:** Conceptualization, Validation, Methodology, Writing – original draft, Writing – review & editing, Funding acquisition. **Yunru Wei:** Investigation, Formal analysis, Visualization, Writing – original draft, Writing – review & editing. **Xilin Jin:** Validation, Investigation, Formal analysis, Data curation, Writing – original draft. **Xucong Lv:** technical support, Visualization, Writing – review & editing. **Zhibin Liu:** Methodology, technical support, Funding acquisition. **Li Ni:** Validation, Project administration, Supervision, Funding acquisition.

## Declaration of competing interest

The authors declare that they have no known competing financial interests or personal relationships that could have appeared to influence the work reported in this paper.
