# Supplementary material for: Spoilage of tilapia by Pseudomonas putida with different adhesion abilities
Source: Curr Res Food Sci. 2022 Apr 11;5:710–7. doi: 10.1016/j.crfs.2022.04.002 (PMC9035656; doi:10.1016/j.crfs.2022.04.002)
Supplement: Multimedia component 1 [file mmc1.docx]

**Table A GenBank accession number of four strains of *Pseudomonas putida***

| Strain | GenBank accession number |
| --- | --- |
| PF01 | OL597894 |
| LP-3 | OL597895 |
| LP-4 | OL597896 |
| LS-6 | OL629391 |

**Table B Volatile components in fish after 0 d and 8 d cold storage**

| Type | Compounds | The content of 0-d cold storage×10^-3^（μg/g） | | | | | The content of 8-d cold storage×10^-3^（μg/g） | | | | |
| --- | --- | --- | --- | --- | --- | --- | --- | --- | --- | --- | --- |
|  |  | Control | LP-3 | LP-4 | LS-6 | PF01 | Control | LP-3 | LP-4 | LS-6 | PF01 |
| Alkanes | Dodecane | - | 2.74 | 2.69 | - | - | 9.04 | 1.29 | 3.55 | 9.81 | 4.79 |
|  | Tetradecane | 3.85 | - | 1.05 | - | - | 46.7 | - | 3.12 | - | - |
|  | Pentadecane | 7.42 | 6.13 | 2.62 | 4.17 | 1.56 | 28.4 | 2.19 | 5.23 | - | 11.8 |
|  | Hexadecane | 3.11 | 2.03 | 0.935 | 1.1 | - | 22.1 | 0.744 | 2.23 | 8.6 | 2.92 |
|  | Heptadecane | - | 7.21 | 7.58 | 4.13 | - | 15.8 | 5.49 | 2.34 | 15 | 3.04 |
|  | Pentadecane, 2,6,10,14-tetramethyl- | 3.41 | 2.08 | - | - | - | 17.2 | - | - | - | - |
|  | Tridecane | - | - | 1.62 | 1.34 | - | - | - | - | - | - |
|  | 1,3-Cyclooctadiene | - | - | - | - | - | - | 1.44 | 4.29 | 3.15 | - |
| Alcohols | Methanethiol | - | 6.88 | - | - | - | - | - | - | - | - |
|  | 1-Butanol, 3-methyl- | - | 15.6 | 12.9 | - | - | - | 7.47 | - | 5.4 | 11.1 |
|  | Phenylethyl Alcohol | - | 4.7 | 7.16 | - | 1.17 | - | 3.02 | 6.52 | - | - |
|  | 1-Octen-3-ol | 44.2 | - | 1.43 | 2.41 | 1.93 | 44.2 | 9.22 | 10.8 | 10.6 | 12.3 |
|  | 1-Octanol | - | 1.48 | 0.751 | - | - | - | 1.33 | 2.83 | - | - |
|  | Benzyl alcohol | - | - | - | - | - | - | - | - | - | - |
|  | Ethanol | - | - | - | - | - | - | 80.4 | 66.8 | 73.5 | 58.6 |
|  | 1-Propanol, 3-(methylthio)- | - | - | 1.14 | - | 0.499 | - | 0.645 | 1.71 | - | - |
| Aldehyde | 1-Hexanol, 2-ethyl- | - | 1.52 | 0.677 | 1.36 | - | - | 0.661 | 10.4 | - | - |
|  | Tetradecanal | 0 | 0 | 0 | 0 | 0 | - | 1.32 | 3.51 | - | 1.96 |
|  | Nonanal | - | 3.57 | 14.5 | 2.6 | 2.48 | 53.1 | 6.47 | 11.9 | 12.4 | 16.4 |
|  | Pentadecanal- | - | - | 15.3 | - | - | - | 1.48 | 3.39 | 2.05 | 3.8 |
|  | Benzaldehyde | 23.3 | 1.76 | 0.887 | 1.5 | 0.615 | 47.3 | 2.73 | 6.92 | 6.33 | 4.06 |
|  | Heptanal | - | - | - | - | - | - | - | - | - | 7.44 |
|  | Hexadecanal | - | 54.02 | 12.3 | 16.5 | 0.929 | - | - | - | - | - |
|  | Benzeneacetaldehyde | - | - | - | - | - | - | - | 4.31 | - | - |
|  | Butanal, 3-methyl- | - | - | - | - | - | - | - | 23.2 | - | - |
| Ketones | Hexanal | - | - | - | - | - | - | - | 38.6 | - | 48.3 |
|  | 5,9-Undecadien-2-one, 6,10-dimethyl-, (E)- | - | - | 1.07 | - | - | - | - | - | - | - |
|  | 2-Nonanone | - | 0.813 | - | 13.3 | - | - | - | 1.67 | - | - |
|  | 2,3-Octanedione | 6.12 | - | - | - | - | 1.98 | - | 3.8 | - | - |
|  | 2-Tridecanone | - | 4.24 | 0.683 | - | - | - | - | - | - | - |
|  | 2-Pentadecanone | - | - | 0.885 | - | - | - | - | - | - | - |
|  | Acetoin | - | - | - | - | - | - | 1.33 | - | - | - |
| Esters | Acetophenone | - | - | - | - | - | - | 0.63 | - | - | - |
|  | Formic acid, hexyl ester | - | 1.96 | - | - | - | - | - | - | - | - |
|  | Hexyl chloroformate | - | 1.95 | - | - | - | - | - | - | - | - |
|  | Hexadecanoic acid, ethyl ester | - | 4.21 | 8.85 | 9.92 | 3.4 | - | 3.94 | 1.97 | - | - |
|  | 9-Octadecenoic acid, methyl ester, (E)- | - | 1.2 | - | - | - | - | - | - | - | - |
|  | Ethyl Oleate | - | 1.85 | - | 2.63 | - | - | - | - | - | - |
|  | Tetradecanoic acid, ethyl ester | - | - | 1.48 | 4.53 | - | - | - | - | - | - |
|  | Formic acid, octyl ester | 3.83 | - | - | - | 7.52 | - | - | - | - | - |
|  | Pentanedioic acid, dimethyl ester | - | - | - | - | - | - | 4.69 | 1.11 | - | - |
|  | Hexanedioic acid, dimethyl ester | - | - | - | - | - | - | 7.18 | 1.77 | - | - |
|  | n-Caproic acid vinyl ester | - | - | - | - | - | - | - | - | - | 14.1 |
| Aromatics | Dibutyl phthalate | - | - | - | - | - | - | 2.54 | - | - | - |
|  | Benzene ,1,3-bis(1,1-dimethylethyl) | 74.8 | 58.1 | 30.4 | 40.4 | - | - | 53.7 | 183 | 184 | 161 |
|  | Naphthalene | - | - | - | - | 2.95 | 0.855 | 2.47 | 5.13 | 5.56 | 6.85 |
|  | Phenol | - | 96.4 | 49.6 | 45.6 | 17.1 | 13.9 | 40.8 | 70.7 | 30.8 | 12.9 |
|  | Phenol, 2,4-bis(1,1-dimethylethyl)- | - | 70.6 | 60.4 | 93.6 | 26.6 | - | 29.5 | 70.4 | 37.4 | 63.3 |
| Acids | 9-Octadecenoic acid, (E)- | 35.5 | - | - | - | - | - | - | - | - | - |
